# Feasibility and Barriers of an Olfactory Training Intervention

**DOI:** 10.1093/geroni/igab046.3065

**Published:** 2021-12-17

**Authors:** Tomiko Yoneda, Natalia López-Bago Cid, Nathan Lewis, Michael Willden, Anna Nelson, Nadia Semenoff, Andrea Piccinin, Jamie Knight

**Affiliations:** 1 University of Victoria, Victoria, British Columbia, Canada; 2 University of Calgary, Calgary, Alberta, Canada

## Abstract

Olfactory dysfunction is a common issue in late-life and can be an early indicator for neurodegenerative diseases. Further, olfactory interventions not only improve olfaction but have shown promise for the delay and treatment of dementia. This study aimed to better understand the feasibility and barriers of implementing an olfactory intervention. Participants (N=23) between the ages of 52-86 (mean=71) years were recruited from the community. A demographic questionnaire showed participants were all non-smokers and identified as women (70%), men (26%), and transgender (4%). The majority were married (61%), while some were separated or divorced (17%), widowed (13%), or single (9%). Four focus groups, guided by both structured and open-ended questions, were conducted and audio-recorded with 3-7 unique participants per group. Data were transcribed, thematically analyzed, and independently coded, which resulted in three overarching themes: (1) cognitive, genetic, and environmental factors of smell, (2) methods to reduce barriers and increase the feasibility of an intervention, and (3) flexibility with technology use. Findings suggest that implementing an olfactory intervention is feasible and of interest to older populations especially when provided with detailed training protocols that have flexibility in the amount of technology used within the study. Barriers included sensitivity to smells, allergies, and dexterity issues. Reducing these barriers will facilitate implementation and decrease the likelihood of attrition. Consulting the target population provides insights into barriers, participant interest, and can assist with the development of training and intervention programs.

